# Genome-Wide Transcriptional Profiles during Temperature and Oxidative Stress Reveal Coordinated Expression Patterns and Overlapping Regulons in Rice

**DOI:** 10.1371/journal.pone.0040899

**Published:** 2012-07-16

**Authors:** Dheeraj Mittal, Dinesh A. Madhyastha, Anil Grover

**Affiliations:** 1 Department of Plant Molecular Biology, University of Delhi South Campus, New Delhi, India; 2 BIOBASE Databases India Private Limited, Bangalore, India; Centro de Investigaciõn y de Estudios Avanzados del IPN, Mexico

## Abstract

Genome wide transcriptional changes by cold stress, heat stress and oxidative stress in rice seedlings were analyzed. Heat stress resulted in predominant changes in transcripts of heat shock protein and heat shock transcription factor genes, as well as genes associated with synthesis of scavengers of reactive oxygen species and genes that control the level of sugars, metabolites and auxins. Cold stress treatment caused differential expression of transcripts of various transcription factors including desiccation response element binding proteins and different kinases. Transcripts of genes that are part of calcium signaling, reactive oxygen scavenging and diverse metabolic reactions were differentially expressed during cold stress. Oxidative stress induced by hydrogen peroxide treatment, resulted in significant up-regulation in transcript levels of genes related to redox homeostasis and down-regulation of transporter proteins. ROS homeostasis appeared to play central role in response to temperature extremes. The key transcription factors that may underlie the concerted transcriptional changes of specific components in various signal transduction networks involved are highlighted. Co-ordinated expression pattern and promoter architectures based analysis (promoter models and overrepresented transcription factor binding sites) suggested potential regulons involved in stress responses. A considerable overlap was noted at the level of transcription as well as in regulatory modules of differentially expressed genes.

## Introduction

Rice is the most important world food crop as more than 3.5 billion people depend on rice for more than 20% of their daily calories (http://irri.org/about-rice/rice-facts/rice-basics). Rice cultivation is carried out in a wide range of ecological environments that have varying altitudes, climates and different soil types. Abiotic stresses (i.e. drought stress, salt stress, flooding stress, oxidative stress and temperature stress) profoundly affect rice cultivation. The severity of most abiotic stresses is on the rise due to intense cultivation practices and environmental deterioration caused by the greenhouse effect.

Temperature extremes drastically affect cultivation of rice crop. Rice is a chilling-sensitive plant [1]. Poor germination, delayed seedling emergence stunted growth and leaf discoloration are some of the notable effects of cold stress (CS) on vegetative growth of rice. During the reproductive stages, degeneration of panicle tip, incomplete panicle exertion, delayed flowering, failure of dehiscence and fertilization, high spikelet sterility and irregular maturity are commonly observed in CS conditions [2]. CS treatment during the flowering stages of rice plant causes abnormal digestion of starch in mature pollen grains, which reduces pollen viability. Rice seed germination is drastically reduced in response to heat stress (HS). HS tends to reduce vigor of rice seedlings and cause abnormal branching patterns of roots [3]. The rate of plant development increases with HS and as a result the duration of developmental phases declines as temperature rises [4]. In rice, number and height of tillers and tillering duration is severely reduced in response to HS [3]. Rice is most susceptible to heat injury during flowering, as pollen viability is particularly sensitive to HS. Even 1–2 h of high temperature at anthesis results in high spikelet sterility [5]. The duration of grain filling in rice is highly sensitive to elevated temperatures. The grain yield of rice is reported to drop by 10% for every 1°C increase in growing season minimum temperature in the dry season [6]. This indicates that decreased rice yields are associated with increased night time temperature which is a result of global warming [6].

While efforts are underway for generation of transgenic rice with enhanced CS tolerance [7]–[10] as well as HS tolerance [11], [12], production of cold and heat tolerant rice that can withstand field-level stress remains elusive. To intensively pursue this goal, there is an urgent need to characterize the physiological processes, biochemical enzymes, molecular mechanisms, and proteins and genes that impart temperature stress tolerance. In general, plants have evolved diverse mechanisms to react to the imposition to stresses. The strategies adopted by plants to combat stress depend on the ecology, timing, severity and the stage of crop growth [13]. Enhancement in reactive oxygen species (ROS) levels is noted under different stress conditions (UV exposure, wounding, pathogen attack, flooding, drought, salt, heat stress and cold stress) as well as by combination of these stresses with high light irradiance [14]–[17]. The levels and kinds of ROS accumulated during stress conditions and their subcellular source determines the expression pattern of specific genes and the induction of stress responsive pathways [16], [18]. Extensive interactions have been noted between HS and oxidative stress (OS) molecular pathways [19]–[23]. During HS, plants activate mechanisms and pathways to control the ROS and redox homeostasis [24]. In *Arabidopsis*, ROS homeostasis has been shown to strongly influence regulation of cold responsive genes. Cold stress binding factors (CBFs) have been shown to be involved in ROS detoxification [25]. Cheng *et al.* [26] has shown a ROS-mediated regulatory module that function as an early component of the chilling stress response pathway in rice plant. Thus, ROS may be referred as a point of convergence between various stress responsive gene networks [27]. Further, an overlapping induction or repression patterns of gene expression is noted in response to different stress conditions, indicating that a common set of signal transduction components may be involved. The transcriptional regulatory networks are important in response to abiotic stress in *Arabidopsis* and grasses [28], [29]. Transcription factors (TFs) are the master regulators owing to the capacity of single TF controlling expression of many target down-stream genes. Nakashima *et al.* [30] have suggested the role of DREB1/CBF; AREB/ABF and NAC regulons in rice, regulating dehydration, salinity, and temperature stress responsive gene expression. Mittal *et al.* [31] have proposed rice HSFs as an important node of cross-talk. The convergence of stress signaling networks to a common set of TFs and regulons suggest the existence of upstream regulatory genes that control plant responses to multiple stresses [32]. Co-ordinated regulation of functionally related genes involved in similar molecular, genetic, biochemical or physiological processes are provided by TFs. The binding of the TFs and cis-element combinations provide a structural basis for the generation of unique patterns of gene expression.

Global transcription profiling using microarray is a powerful approach to understand the molecular aspects under different stress conditions. In rice, gene expression profiles during abiotic stresses have been analyzed in response to different agents [26], [33]–[39]. However, the data from most of these studies can not simply be compared because of the differences in the conditions under which the respective experiments have been performed e.g. age of the plants, light regime, growth conditions, stress treatments, microarray platform and the analysis criteria used. This study presents transcription profile of rice seedlings in response to CS, HS and OS treatments in a single genotype, thus providing a platform for comparison of the various genes and regulons up-regulated following the three stress conditions. Two different time points were used in our experiments to score ‘primary’ (at early time points) and ‘secondary’ (at late time points) changes in the gene expression. Composite module analyst (CMA) analysis was employed to unravel the key transcription factors that may explain concerted expression changes of specific components in various signal transduction networks.

It is important to note that the levels of different stresses (i.e. CS, HS and OS) employed in this study are largely sub lethal. Exposure to sub lethal stress has been shown to bring about the required changes in the plant metabolism necessary for withstanding the subsequent severe stress levels (i.e. cross-proection phenomenon) [40]–[43]. Based on this hypothesis, we propose that the gene expression changes noted in this study might be important in providing the adaptive advantage to survive under lethal stress. This study highlights the common as well as specific aspects of the transcriptional changes following CS, HS and OS. Our data suggests that a variety of TFs besides the well-known regulons govern the CS, HS and OS responses. This study reveals that the ROS mediated common regulatory modules are involved in the response of rice seedlings to temperature extremes.

## Materials and Methods

### Growth of Rice Seedlings, Stress Treatments

Rice [*Oryza sativa* ssp. *indica* L; cultivar Pusa Basmati (PB1)] seeds were germinated as described before [31]. Germinating seeds were supplemented with rice growth medium [44]. After 7d, seedlings were transferred to 100 ml glass beakers and allowed to grow further, to ensure that seedlings recover from injury to the roots taken place due to uprooting from the cotton bed. On d9, beakers containing seedlings were transferred to growth chamber (light intensity ∼80 µmol m^−2 ^s^−1^; humidity 40–50%; temperature 28±2°C; light regime 14 h light/10 h dark cycle). The stress conditions were standardized, with an emphasis on the uniformity of the growth and stress regimes to minimize the occurrence of secondary stresses during the treatment. The plants employed for stress treatments were grown in uniform conditions to minimize the physiological differences between individual plants, as far as possible. Similar growth conditions (light intensity, humidity, supplementation of the media, size of the beakers etc.) were maintained for each stress treatment. For temperature stress, uniform-sized seedlings (10 d old) were transferred to beakers, which contained distilled water at 42±1°C for heat stress (HS), at 5±1°C for cold stress (CS), and 10 mM H_2_O_2_ at 28±2°C for oxidative stress, and maintained at the requisite temperatures in BOD (for different time intervals as shown). For oxidative stress 10 mM H_2_O_2_ was used (H_2_O_2,_ a stable ROS, has long half-life; [45]). Subsequent to completion of the stress intervals, tissues were harvested (whole seedlings were pooled to have ∼100 mg tissue and taken as one biological replicate), frozen in liquid nitrogen and kept at −80°C. Non-stressed plants for control were handled exactly in similar manner. All rice seedlings harvested post stress treatment was grown at the same time. RNA samples from three independent biological replicates for stressed and control tissues were then processed for microarray hybridization.

### Microarray Analysis and Q-PCR

60 mer rice 44 k oligo DNA array Kit (AMADID: No: 015241, Agilent Technologies) which contains 45018 features/microarray and ∼40000 transcripts was used. Total RNA was isolated from ∼100 mg tissue using Tri-reagent (Sigma, USA) as per the manufacturer’s instructions, and further purified using RNA easy mini elute kit (Qiagen, USA). The yield and RNA purity were determined spectrophotometrically. Integrity of the RNA was checked using Agilent Bioanalyzer (Agilent Technologies, USA). 200 ng total RNA was labeled with Cy3 using an Agilent low RNA input fluorescent linear amplification kit (Agilent Technologies, USA). Hybridization and wash processes were performed according to the manufacturer’s instructions and hybridized microarrays were scanned using Agilent microarray scanner (G2505B, Agilent Technologies, USA). Feature extraction software (version 9.5.1 Agilent Technologies, USA) was employed for the image analysis and data extraction process. The normalization was done using GeneSpring GX version 7.3.1 (Agilent Technologies, USA) using the recommended per chip and per gene data transformation: set measurements less than 0.01 to 0.01, per chip: normalize to 50th percentile per gene: normalize to specific samples (treated vs control). Data analysis was done using GeneSpring GX version 7.3.1 (Agilent Technologies, USA) and Microsoft Excel. Three biological replicates were used in the microarray analysis. There was a good correlation among the three biological replicates of a condition as evident by the fact that more than 80% of the probes in the microarray showed variance <0.25% among the biological replicates when calculated for the complete data and showed good overall correlation coefficient values. PCA plots among the biological replicates are provided in the Fig. S1. The data discussed in this publication have been deposited in NCBI’s Gene Expression Omnibus (Mittal *et al.* 2010) and are accessible through GEO series accession number GSE19983 (http://www.ncbi.nlm.nih.gov/geo/query/acc.cgi?acc=GSE19983). We applied the criteria of at least 2.0 fold change (Log2 values) in gene expression levels and p-value revealed by t-test of less than 0.05. Multiple testing correction (Benjamini and Hochberg False Discovery Rate multiple testing correction) was applied on the t-test p-values and these corrected p-values were used to identify the significantly changed genes. The RAP-DB IDs given in the results corresponds to the IRGSP genome build 4 (http://rapdb.dna.affrc.go.jp). Q-PCR was carried out as described earlier [31]. Two biological replicates and three technical replicates were used for the Q-PCR analysis. cDNA for the real-time reactions were synthesized using the same RNA samples that were used for microarrays.

### CMA and F-Match Analysis

Construction of composite promoter models (CPM) as combinations of closely localized TF-binding sites in promoters were identified with CMA software using the ExPlain™ Plant 3.0 (BIOBASE GmbH) in the promoters of differentially expressed genes during the three stress conditions. Promoter analyses were carried for up-regulated or down-regulated genes (Yes set) with random set of 500/1000 genes (No sets were extracted from rice promoters dataset present in ExPlain™ Plant software) based on the number of Yes set genes. Computational identification of TF binding sites in the promoter sequences under study was done with Match™ program which applies the full TRANSFAC library of Positional Weight Matrices (PWM) from TRANSFAC® database with added PWM of heat shock proteins of vertebrates as heat shock proteins are highly conserved across species. F-Match and Match analysis were carried for up- or down- regulated genes with a promoter window of −500 to +100 bp around TSS (Transcription Start Site). In F-Match analysis, p-value <0.01 was chosen and further in the results, the PWM with ratio >1.3 Yes/No frequencies and Matched promoter p–value <0.05 was selected. CMA analysis were carried with results of Match analysis with parameters; Genetic algorithm iterations run for 12 h, NC limit “None”, Population size of 200, 3 to 5 TF pairs, in 1 module in 1 group, in 200 bp window size, with optimized distance between the pairs options selected. More details and case studies on the latter aspect can be seen elsewhere [46]–[50].

### Functional and Clustering Analysis

ExPlain™ Plant 3.0 software program (BIOBASE GmbH) was employed to explore statistically over-represented groups in the up- or down-regulated genes. Plant expression conditions, Plant ontologies and gene ontology (GO) classification were carried with p-value <0.05. Cluster analysis were carried for up- or down- regulated genes with rice protein interaction dataset from the ‘Reactome database’ (http://www.reactome.org), taken as secondary set with parameters cluster separation degree as “0” and Distance threshold as “3” value.

## Results

### Gene Expression Profiling in Response to CS, HS and OS in Global Context

**Figure 1:**
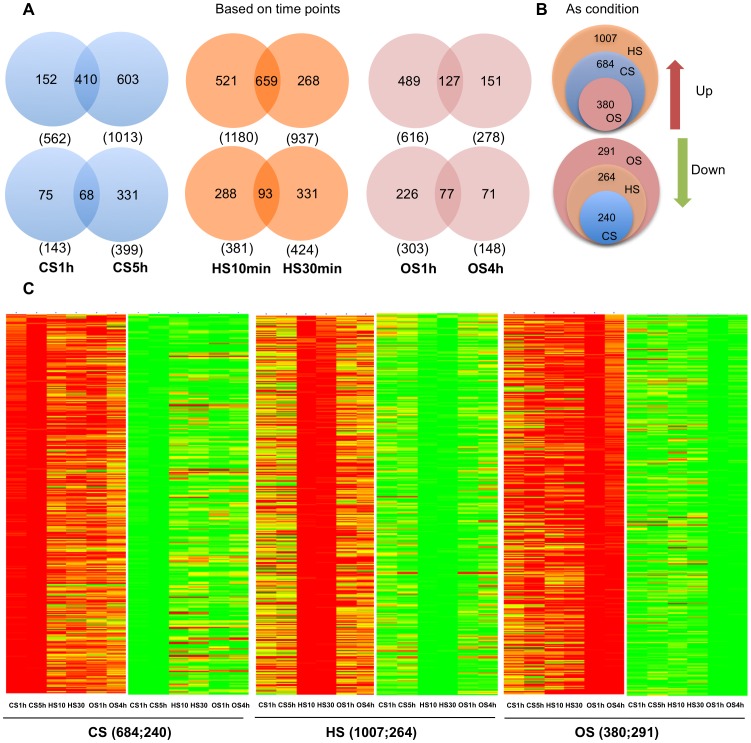
Global gene expression pattern in response to CS, HS and OS. A. Representation of the number of differentially regulated genes following CS (CS1 h and CS5 h), HS (HS10 min and HS30 min) and OS (OS1 h and OS4 h). Numbers given in brackets represent the total number of differentially expressed genes in CS1 h, CS5 h, HS10 min, HS30 min, OS1 h and OS4 h respectively. B. Differentially expressed genes for a stress condition (i.e. the data from the two time points for one stress is considered as a condition). C. Hierarchical cluster image showing differentially expressed genes for stress conditions depicted in 1B. Detailed gene lists are provided in Data S1.

Transcript profiles as affected by CS, HS and OS were analyzed by microarray analysis (Fig. 1A). The comparison of the number of genes differentially regulated following the two time points of each stress condition (early and late) as well as gene common to the two time points are shown in the Venn diagram (Fig. 1A). It is evident that HS and OS response is more pronounced in terms of transcription induction in the early time points, than that of CS response. When the two CS treatments (based on the time points) were taken as a condition CS, 684 genes were up-regulated and 240 genes were down-regulated with respect to control. Similarly, in case of HS, 1007 genes were up-regulated and 264 genes were down-regulated and in OS, 380 were up-regulated and 291 were down-regulated with respect to control (Fig.1 B). Fig. 1C shows hierarchical cluster of differentially expressed genes (DEGs) shown in Fig. 1B. The detailed gene lists are presented in Data S1.

**Figure 2:**
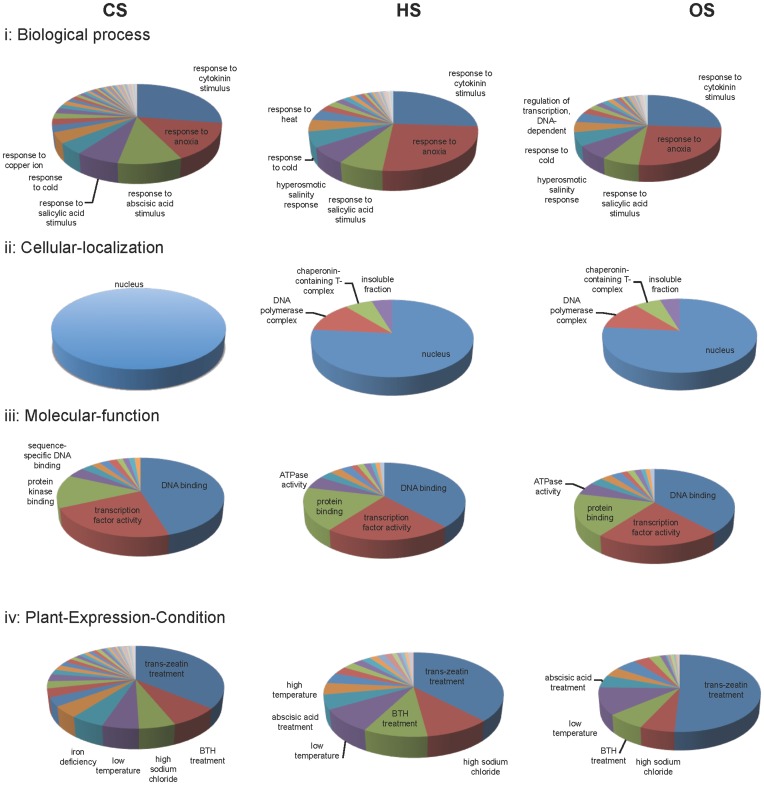
Functional classification of up-regulated genes. Significant GO/functional categories enriched for the up-regulated genes during CS, HS and OS are shown in the form of a pie chart. For details refer to the Data S2.

**Figure 3:**
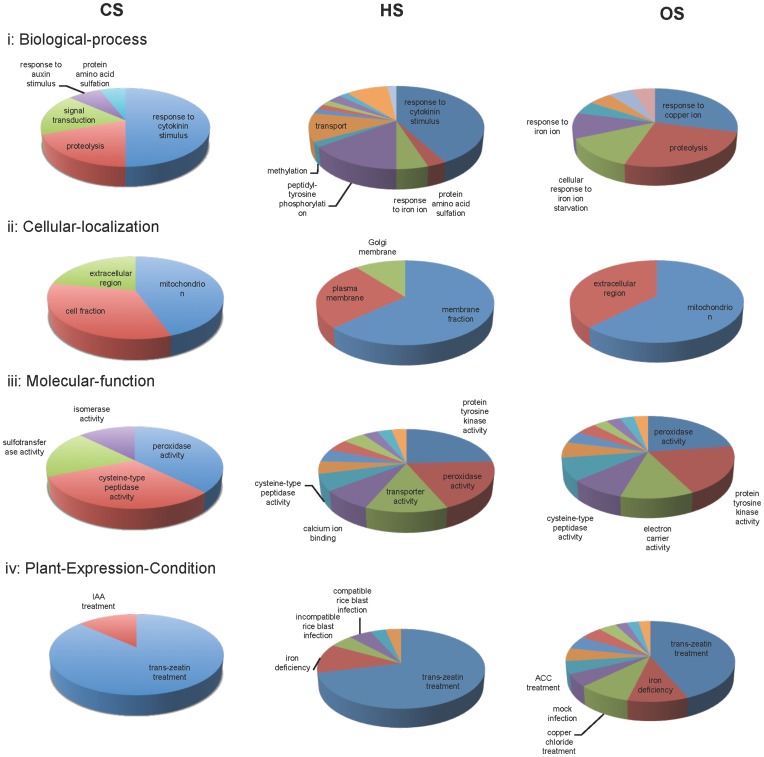
Functional classification of down-regulated genes. Significant GO/functional categories enriched for the down-regulated genes during CS, HS and OS are shown in the form of a pie chart. For details refer to the Data S2.

ExPlain™ Plant 3.0 software program (BIOBASE GmbH) was employed to explore statistically over-represented groups in data sets according to Plant expression conditions, Plant ontologies and gene ontology (GO) classification. Figures 2 and 3 show the significant categories enriched for the DEGs, namely ‘Biological process’, ‘Cellular-localization’, ‘Molecular-function’ and ‘Plant-Expression-Condition’ according to BKL plant database (Fig. 2: up-regulated genes; Fig. 3: down-regulated genes). In addition, DEGs were also classified to other extended classes based on BKL plant database. Various developmental stages were represented when DEGs during CS were classified on the basis of plant ontology (PO) growth stage and plant structure. The enrichment of ‘inflorescence development, reproductive growth and flowering stages, tapetum, pollen development and microspore specific classes’ for the CS up-regulated genes was noteworthy. Grouping of DEGs during HS for GO terms, plant growth stages and structure revealed ‘D pollen mother cell meiosis stage and microspore’ as the top most GO class for up-regulated genes. GO categories associated with reproduction like ‘F mature embryo stage, seed maturation stage, and stamen primordium visible’ were noted. Two trait ontology terms i.e. ‘drought tolerance and drought recovery’ were enriched for HS up-regulated genes. Trait ontology based classification revealed ‘mineral and ion content related trait as well as growth and development trait’ as enriched terms for HS down-regulated genes. Enrichment of inflorescence and its developmental stages, as well as spikelet in the GO term plant ontology growth stage and structure was noted. Classification based on the trait ontology revealed ‘grain yield and mineral ion content related trait’ for the OS down-regulated genes. Detailed functional classification for biological processes, cellular localization, molecular function, plant expression conditions, PO growth stages, PO plant structure and trait ontology are presented in Data S2 for the DEGs during CS, HS and OS.

**Figure 4:**
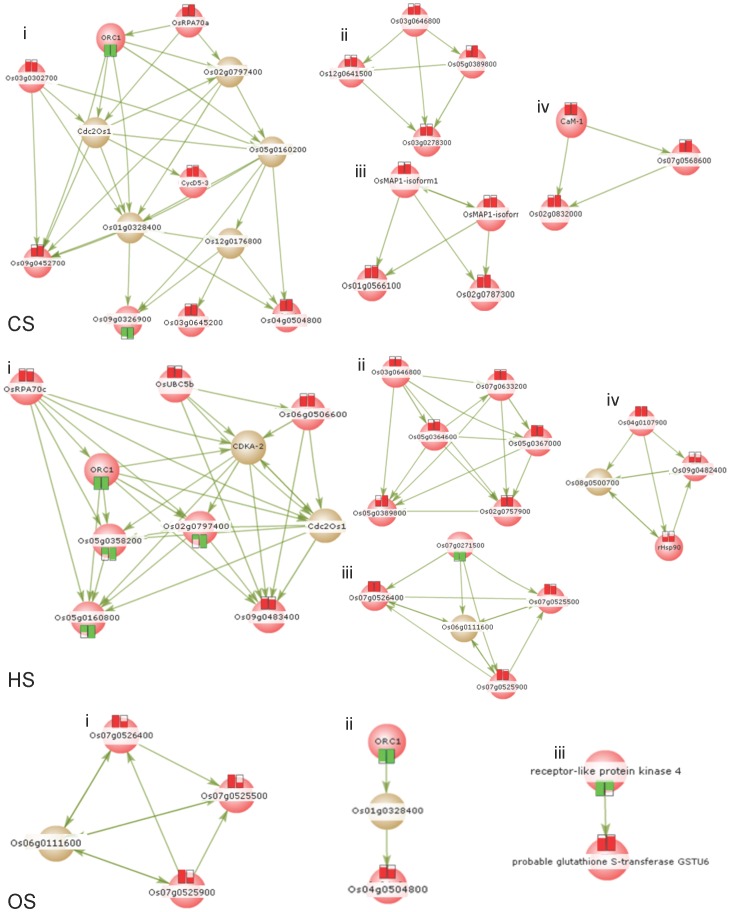
Network analysis. Clusters enriched for DEGs during CS, HS and OS. Hit molecules – red color, Inter connecting molecules - grey color. Red and Green Box – level of expressions during stress treatments respectively. Details of the genes shown in the clusters are provided in Table S1.

Rice protein interaction dataset from the ‘Reactome database’ (http://www.reactome.org) was taken as secondary set to carry out cluster analysis. This analysis identifies common signaling molecules in the vicinity of genes from the input list within the signaling networks. The significant clusters enriched are shown in Fig. 4. Clustering of DEGs from CS treatment yielded networks with DNA transcription (cluster i), RNA metabolism (cluster ii), kinase function (cluster iii), and Ca^2+^-mediated signaling molecules (cluster iv). Similar clustering of DEGs from HS treatment yielded networks with DNA transcription (cluster i), RNA metabolism (cluster ii), chalcone synthase metabolism (cluster iii), and heat shock proteins (HSPs) (cluster iv). In case of OS treatment, networks with chalcone synthase metabolism (cluster i) and two more clusters were noted. The physiological, molecular and biochemical processes involving these clusters may be termed as key mechanisms during the respective stress responses. The details of genes shown to be the part of network/clusters are given in Table S1.

**Figure 5:**
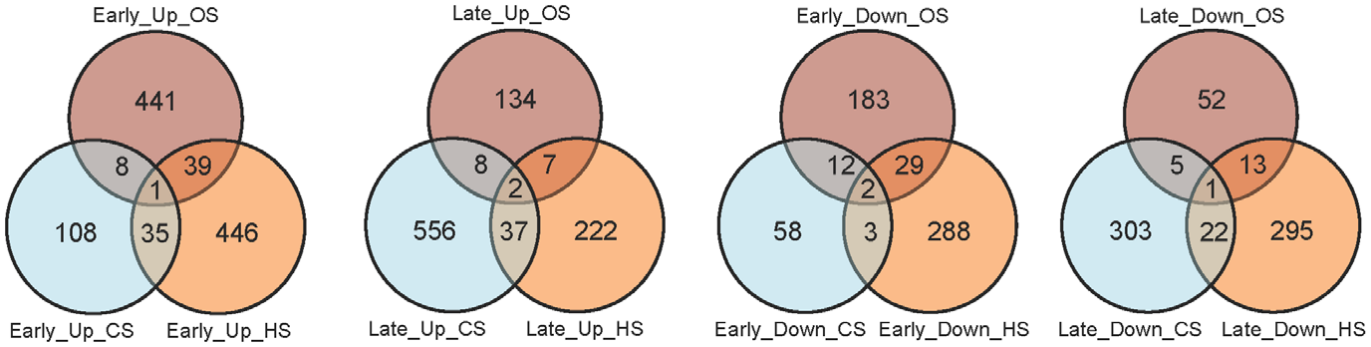
Specific gene expression pattern in response to CS, HS and OS. Representation of the number of DEGs based on the time points of stress treatments is depicted classifying the number of genes in early or late responsive genes. Early refers to time points: CS1 h, HS10 min and OS1 h of CS, HS and OS stress condition. Late refers to time points: CS5 h, HS30 min and OS4 h of CS, HS and OS stress condition. Detailed gene lists are provided in Data S1.

Detailed analysis was undertaken by splitting genes into gene lists having genes with specific expression profile under the given stress, or the genes, which are common to two or three stresses. In order to identify early or late responsive genes, the salient findings are analyzed with reference to time points (i.e. early or late) for each stress type (Fig. 5). Detailed gene lists (with fold changes, GO, gene descriptions, Gramene pathways and other relevant information) are provided in the Data S1. Significant classes of genes i.e. kinases, transcription factors, signal transduction components, genes involved in ubiquitin protein ligase reaction, auxin responsive genes and metabolism related genes noted in the group of DEGs are highlighted in Table 1. Selective gene expression changes are highlighted in the following sections.

**Table 1:** Significant classes (shown underlined) of genes i.e. kinases, transcription factors, signal transduction components, genes involved in ubiquitin protein ligase reaction, auxin responsive genes and metabolism related genes noted in the group of differentially regulated genes.

CS specific	HS specific	OS specific
Protein kinases- (19) [*5*]; TFs & STCs- WRKY(4), F-box (3), NAC (4), Myb (5) [*3*], Zn-finger(7) [*2*], HSFs (2), DREB (2), ERF (3);Ubiquitin protein ligase rxn- (7) [*3*];Metabolism related- (sucrose, stachyose,terpene, trehalose-6 phosphatase)synthase; Auxin responsive- OsSAURs (2)	Protein kinases- (5); TFs & STCs- HSFs (6), DREB (4), Zn-finger(5), bZIP (3), WRKY (2), MADS box (2), NAC (2) AP2 domaincontaining (2), MYB (1) [*1*] *,* PP2C (3), Auxin response factor23 (1) *EREBP * [*1*] *, TSRF * [*1*] *;* Ubiquitin protein ligase rxn- (9);Metabolism related- (galactinol, terpene, stachyose, chalcone,inositol-3-phosphate, strictosidine, digalactosyldiacyl glycerol) synthase; Auxin responsive- (0)	Protein kinases- (5) [*1*] *;* TFs and STCs- HSFs (2), F-box (2), Zn-finger (3), HLH DNA binding domain containing (1), EREBP (1), Ethylene responsive TF (1), *MYB2 * [*1*]; Metabolism related- trehalose-phosphate rxn (1); Auxin-responsive *OsSAUR31*
**Early CS**	**Early HS**	**Early OS**
Protein kinases- (3) [*3*] *;* TFs & STCs- F-box(2), NAC (2), MADS- box (1) DREB (1),sbCBF6 (1), *Myb * [*2*] *, HLH DNA* *binding protein * [*1*] *; U* biquitin protein ligase rxn- (7) [*3*]; Metabolism related- (terpene,sucrosephosphate, pseudourylate) synthase;Auxin responsive OsSAUR26, auxin response factor 23, *OsSAUR28*	Protein kinases- (17) [*13*]; TFs & STCs- HSFs (6), Zn fingerprotein (7), WRKY (12), F-box (4) [*6*], NAC (1) [*2*], Myb (2) [*1*],Zn-finger (7) [*5*], HSFs (6), DREB (1), G-box binding (1),MADS-box (13), PP2C (2) Ethylene receptor-1 (1), ERF1 (1) [*1*],Baby Boom2 (1), Argonaute (1) *HLH domain containing * [*2*] *,* *bZIP * [*1*] *, EREBP * [*1*] *, Ethylene receptor protein * [*1*] *,* *Leucine Zipper * [*1*] *, LRR * [*1*] *;* Ubiquitin protein ligase rxn- (16) [*5*]; Metabolism related- (chalcone,stachyose) *[spermidine, falavonol, terpene, strictosidine,* *sucrose]* synthase; Auxin responsive-*OsSAURs (3), auxin repressed protein gene (1),* *auxin metabolism related genes (2)*	Protein kinases- (20) [*12*]; TFs & STCs- WRKY (11), F-box (2), NAC (2), Myb (4) [*1*], HSFs (4), HLH DNA binding domain containing (2) [*1*], AP2 domain containing (5), DREB (1), ZIM (4), bZIP (2), DRE binding factors (2), NAM-like (1), leucine zipper (1; ATHB-6), EREBP (1), Zn*-finger * [*3*] *;* Ubiquitin protein ligase rxn- (11 includes Zn-finger C3HC4 (2), H2 finger (2), Zn-finger (1)) [*3*]; Metabolism related- (trehalose 6-phosphate synthesis (3), (galactinol, phytoene, CTP, stachyose, cellulose, chalcone, flavonol, terpene, strictosidine, glutamate, granule bound starch) *[chalcone, flavonol, glutamate, granule bound starch] synthase;* Auxin responsive- (0)
**Late CS**	**Late HS**	**Late OS**
Protein kinases- (32) [10]; TFs & STCs-WRKY (8) [*1*], F-box (8) [*2*], NAC (6) [*1*],AP2 domain containing (2), HSFs (4) [*1*],DREB (2), bZIP (1), Myb (1) [*1*], ZIM (1),PP2C (5), PHD-finger (3), OCS-element bindingfactor (1), CONSTANS protein (1), Ethyleneresponsive TF (1), EREBP-4 likeprotein (1), *Zn finger * [*2*] *, MADS-box * [*1*] *;* Ubiquitin protein ligase rxn- (11) [*5*];Metabolic reactions- (stachyose, phytoene, cellulose, CTP, sucrose, terpene)synthase; Auxin responsive- OsIAA9, auxinresponse factor 16, auxin-inducibleprotein gene	Protein kinases- (5) [*14*] *;* TFs & STCs- WRKY (2), F-box (2) [*1*],NAC (3) [*1*], Myb (4) [*3*], Zn-finger (7) [*2*], HSFs (1),MADS-box (1), DREB (1), AGO protein (1), *PP2C * [*1*] *;* Ubiquitin protein ligase rxn- (7) [*3*] *;* Metabolism related- (genesinvolved in trehalose 6 synthase rxn (2), myo-inositol-1-phosphate synthase rxn (1) terpene synthase (2)*chalcone synthase 8;* Auxin responsive- *OsSAURs (1)*	Protein kinases- (5) [*1*]; TFs & STCs- AP2 domain containing (1), *WRKY * [*2*] *, Myb * [*1*] *;* Ubiquitin protein ligase rxn- (2); Metabolism related- (trehalose 6-phosphate synthesis (3), (galactinol, phytoene, CTP, stachyose, cellulose, chalcone, flavonol, terpene, strictosidine, glutamate, granule bound starch), *[chalcone, flavonol, glutamate, granule bound starch] synthase;* Auxin responsive- (0)

Numbers in () are for up-regulated genes, and in *[]* are for down-regulated genes. The italicized text is used for down-regulated genes. For details refer to Data S1. rxn; reaction.

### Transcript Expression Profiling Specific to CS

During cold, 326 genes were exclusively (i.e. noted only in CS and not in HS and OS) up-regulated by more than two folds. Genes involved in redox homeostasis like dehydrogenase and copper chaperone for SOD were found up-regulated. A significant number of genes that may be part of Ca^2+^ signaling were up-regulated. A variety of transporters were also noted. On the other hand, 47 genes were specifically down-regulated during CS. In addition to the TFs and kinases (Table 1), transcripts of expansin protein (causes loosening and extension of plant cell walls; Os05g0277000) and dynamin protein (involved in cell membrane severity; Os08g0425100) were found repressed. Further, peroxidase 1 precursor gene (Os07g0639000) and genes that may be involved in detoxification and redox homeostasis like oxidases, endohydrolase and sulfo transferse were noted. The numbers of DEGs described in the above section were derived using the number of DEGs during CS as a condition (Fig. 1B).

Specific aspects related to gene expression changes induced by CS are further taken herein as CS1 h and C55 h. The respective lists represent the genes that are specific to CS i.e. not found during HS and OS, at early and late time points respectively (Data S1). 108 genes specifically up-regulated early in the CS represent the early responsive genes. Besides various TFs and kinases (Table 1), genes involved in Ca^2+^ signaling were noted in this group of genes. 58 genes were found specifically down-regulated at CS1 h. In addition to the TFs and kinases (Table 1), antiporters, transporters and aquaporins were specifically repressed.

On the other hand, 556 genes were found exclusively up-regulated during CS5 h representing the late responsive genes during cold stress. Several genes that may have role in Ca^2+^ mediated signaling cascade were noted in this group. Up-regulation of chaperone genes highlights the role of HSPs in CS. Genes involved in redox homeostasis like glutathione S-transferases (GSTs), glutaredoxin II member, cytochrome P450 gene family members and genes involved in peroxidase reaction were noted. 303 genes were down-regulated exclusively at CS5 h. Six genes coding for different transporters and 7 genes of cytochrome P450 gene family were noted. sHSPs (encoding proteins of 16.9 kDa and 17.5 kDa) were found exclusively down-regulated at this time point.

### Transcript Expression Profiling Specific to HS

555 genes were exclusively up-regulated during heat stress. These included several genes which are well-characterized components in HS responsive regulon. Genes involved in Ca^2+^ signaling like calnexin, calreticulin and EF hand family protein were up-regulated. Up-regulation of different synthase genes and various transporter genes was noted. 82 genes were exclusively down-regulated during HS. Among these genes dehydrogenases, genes involved in peroxidase reaction, various transporter genes and an antiporter protein gene are noteworthy. The numbers of DEGs described in the above section were derived using the number of DEGs during HS as a condition (Fig. 1B).

Specific aspects relating to gene expression changes induced by HS are further taken here as early and late changes during HS (Data S1). At HS10 min, 557 genes were specifically up-regulated. These included genes that are well-characterized components in HS responsive regulon. Genes involved in Ca^2+^ signaling and redox homeostasis were noted in this subgroup. Several genes coding for different transporters, and hydrolases were found up-regulated. 288 genes were specifically down-regulated at HS10 min. Among these, down-regulation of a CBL-1 gene, 3 genes coding for phosphatases and 4 genes coding for different transporters was noteworthy. Five genes involved in peroxidase reaction and 6 cytochrome P450 gene family member genes were also down-regulated.

At HS30 min, 222 genes were exclusively up-regulated. In this gene list, one gene each of sHSP (HSP17.4), HSP30, HSP70, HSP90 and a DnaJ domain containing protein were noted. Transcripts of genes involved in Ca^2+^ signaling were up-regulated. Up-regulation of cytochrome P450 gene family members and genes involved in GSH trans-reaction highlights the role of redox homeostasis at HS30 min. A total of 295 genes were exclusively down-regulated during HS30. These included as many as fourteen genes coding for various transporters and 3 genes for antiporters. Three genes involved in Ca^2+^ signaling were also down-regulated.

### Transcript Expression Profiling Specific to OS

Of the 70 genes found exclusively up-regulated during OS, 9 genes codes for GSTs and one gene for glutathione reductase. Several genes associated with the state of redox homeostasis were up-regulated. 59 genes were down-regulated exclusively during OS. Among these, transporter genes were significantly enriched. The numbers of DEGs described in the above section were derived using the number of DEGs during OS as a condition (Fig. 1B).

Specific aspects relating to gene expression changes induced by OS were further analysed as early and late changes during OS (Data S1). A total of 441 genes were up-regulated specifically at OS1 h. Six genes involved in Ca^2+^ signaling and 6 phosphatase genes (including 5 class 2 C members) were noted. Transcript levels of 3 chaperone genes, 7 transporter genes, 6 oxidoreductase genes, and 3 VQ motif family genes were up-regulated. In addition, 13 members of cytochrome P450 gene family were up-regulated. A total of 183 genes were down-regulated exclusively at the early time point OS. Among these, genes involved in redox homeostasis were noted. Transcripts of different transporter genes and genes involved in Ca^2+^ signaling were found repressed at OS1 h.

A total of 134 genes were up-regulated exclusively at OS4 h. Among these, 8 HSP (including sHSPs, HSP100, HSP80 and HSP70) genes were up-regulated. Genes involved in peroxidase reaction, GSH/GST trans-reactions and cytochrome P450 family gene member were noted in this sub group. A total of 52 genes were down-regulated exclusively at OS4 h. Among these, genes involved in peroxidase reaction, cytochrome P450 gene family members, metallothionein–like protein genes, reductases, dirigent like protein pDIR17 gene and dopamine β-monooxygenese gene were important to note.

### Gene Expression Profiling-Common Elements in CS, HS and OS

19 genes were up-regulated by 2 or more folds in all three stress conditions irrespective of the time points (Table S2). Sixty-four genes were common among the up-regulated genes during CS and HS, while 7 genes were down-regulated both in CS and HS. With respect to CS and OS, 10 genes were commonly up-regulated and 14 were down-regulated. 36 genes were commonly up-regulated and 3 genes were down-regulated during HS and OS.

### Regulation of miRNA in CS, HS and OS

Regulation of genes by miRNAs highlights the importance of post-transcriptional gene regulation. In this study, probe sets for several miRNA genes were found differentially regulated (Table S3). Their putative and predicted targets (based on the data by Archak and Nagaraju [51]) are also presented (Table S3). It is noteworthy that some of these miRNAs have genes involved in redox homeostasis (e.g. superoxide dismutase) as their targets. In our data, redox homeostasis related genes were found differentially regulated in all the three stress conditions tested. This supports the view that ROS plays a central role in the abiotic stress response. The functional validation of the stress responsive miRNAs remains as an important endeavor.

### Transcript Profiling of Transcription Factor Genes in CS, HS and OS

**Figure 6:**
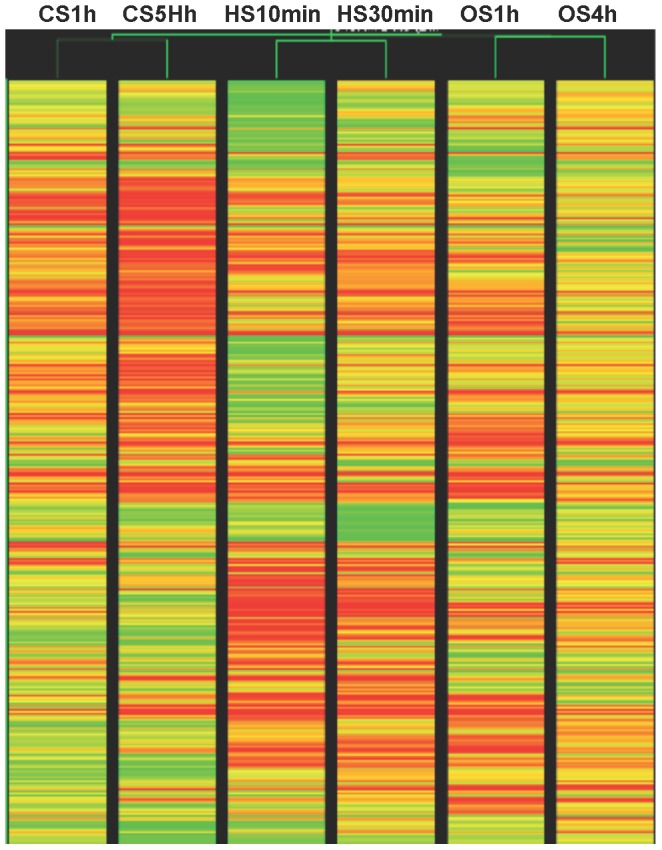
Hierarchical clustering of the differentially expressed transcription factors (TFs). Detailed gene lists (with fold changes, GO, gene descriptions, Gramene pathways and other relevant information) are provided in the Data S3.

The expression profile of all the probes present on the array representing transcription factors in rice (as per rice transcription factor database, Rice TFDB (2.1), [52] was analyzed. The expression of genes expressing differentially by more than or equal to 1 fold (Log2 values, i.e. two times differential change in the transcript abundance with respect to control) with a p-value of <0.05 were considered significantly up- or down-regulated. The above cut-off level was based on the assumption that induction of TFs at low to moderate levels can have significant effects on downstream gene expression. The differential profile of differentially-expressed TF genes (in the form of hierarchical cluster) present on the array is shown in Fig. 6 and the details are provided in Data S3. Specific transcription factors up-regulated by more than one fold in all the three stress conditions at both time points of each stress type are shown in Table 2. Genes encoding for WRKY (Os02g0462800), MYBS (Os02g0618400 and Os05g0442400), CRT/DRE binding proteins (Os02g0677300) and ZIM domain proteins (Os03g0180800, Os03g0180900, Os10g0391400, Os10g0392400) were significantly up-regulated in response to CS, HS and OS. Four gene members belonging to rice ZIM family of TFs were significantly up-regulated in response to the three stress conditions tested. A novel gene Os01g0780800 belonging to SAND domain proteins (SAND domain proteins; Sp100, AIRE-1, NucP41/75 and DEAF-1 are known to be chromatin associated; [53]) showed significant up-regulation. Rice HUA1 (Os01g0914700; HUA1 is a RNA binding protein and is involved in floral patterning) was up-regulated during these stress conditions, with almost 4-folds change during initial cold stress. Seven TF genes were down-regulated in all the stress conditions (Table 2). These genes included 3 Myb/Myb related genes and one gene each coding for a WRKY (Os01g0972800) and ZIM domain containing protein (Os07g0153000). Q-PCR was carried out to validate the microarray expression profile of the selected TFs described above (Fig. S2). A good correlation in the pattern of expression was noted between microarray and Q-PCR data.

**Table 2:** Transcription factor genes noted to be differentially expressed (both up- and down-regulated) during CS, HS and OS conditions by more than one fold.

Up-regulated TFs										
Locus ID	cDNA	CS1H	CS5H	HS10	HS30	OS1H	OS4H		Description	
Os01g0780800	AK108614	*1.65*	*2.93*	*4.98*	*3.84*	*3.44*	*1.82*		SAND domain containing protein.	
Os01g0826400	AY676925	*2.23*	*3.58*	*1.04*	*1.75*	*2.72*	*2.55*		WRKY transcription factor 24.	
Os01g0839100	AY077725	*1.03*	*2.30*	*1.37*	*1.95*	*1.83*	*1.30*		Zn-finger, C2H2 type domain-containing protein.	
Os01g0917400	AK112102	*3.99*	*1.88*	*1.72*	*1.88*	*2.19*	*1.22*		Floral homeotic protein HUA1.	
Os01g0952900	AK069958	*4.51*	*5.39*	*4.81*	*4.62*	*4.97*	*3.08*		Conserved hypothetical protein.	
Os02g0181300	AK058773	*2.23*	*3.05*	*1.73*	*2.05*	*2.39*	*1.20*		WRKY transcription factor 71 (TF WRKY09).	
Os02g0462800	AK110587	*1.77*	*3.70*	*2.04*	*2.71*	*4.15*	*2.91*		WRKY transcription factor 42 (TF WRKY02).	
Os02g0527300	AK101934	*2.94*	*3.71*	*2.83*	*3.69*	*1.01*	*1.76*		Heat shock transcription factor 31 (Fragment).	
Os02g0618400	AK099283	*3.39*	*3.56*	*1.96*	*3.14*	*2.65*	*1.57*		MYB8 protein.	
Os02g0624300	AK112056	*2.46*	*3.31*	*1.78*	*1.84*	*1.98*	*1.06*		MYB1 protein.	
Os02g0676800	CI545726	*6.42*	*6.27*	*3.65*	*3.72*	*2.95*	*1.56*		(No Hit)	
Os02g0677300	AK060550	*5.59*	*4.39*	*2.19*	*5.59*	*2.57*	*3.78*		CRT/DRE binding factor 1.	
Os02g0685200	AK111726	*4.60*	*6.64*	*3.91*	*2.40*	*5.28*	*1.57*		Myb, DNA-binding domain containing protein.	
Os02g0764700	AK107146	*5.38*	*5.61*	*3.91*	*4.17*	*3.81*	*1.10*		Pathogenesis-related TF and ERF domain containing protein.	
Os03g0180800	AK070649	*3.99*	*4.38*	*2.44*	*3.51*	*3.97*	*1.65*		ZIM domain containing protein.	
Os03g0180900	AK073589	*1.76*	*2.84*	*2.46*	*2.26*	*3.05*	*1.80*		ZIM domain containing protein.	
Os03g0191900	CI428634	*1.98*	*4.09*	*3.17*	*2.37*	*4.61*	*1.53*		AP2 domain family TF homolog (ABI4:ABA-insensitive 4)	
Os03g0212300	AK070861	*2.78*	*1.26*	*2.76*	*2.08*	*1.11*	*2.25*		Transcriptional factor B3 family protein.	
Os03g0437200	AK059839	*1.94*	*2.94*	*1.45*	*1.72*	*3.21*	*2.47*		Zn-finger, C2H2 type domain-containing protein.	
Os03g0741100	AK071734	*2.75*	*3.88*	*3.50*	*3.67*	*3.25*	*1.74*		bHLH domain containing protein.	
Os03g0815100	AK067690	*2.57*	*3.25*	*3.00*	*1.60*	*2.80*	*1.21*		OsNAC6 protein.	
Os03g0820300	AK068861	*1.94*	*4.57*	*5.08*	*3.37*	*4.89*	*3.10*		ZPT2-14.	
Os03g0820400	AK119690	*3.32*	*4.47*	*3.53*	*2.60*	*3.90*	*2.15*		ZPT2-13.	
Os04g0301500	AB040744	*3.13*	*3.86*	*5.45*	*3.86*	*4.14*	*1.01*		bHLH domain containing protein.	
Os04g0399800	CI145320	*1.65*	*2.86*	*1.29*	*1.00*	*4.17*	*1.53*		Pathogenesis-related genes transcriptional activator PTI5.	
Os05g0442400	AK107134	*1.05*	*3.89*	*5.65*	*4.46*	*5.82*	*2.00*		Transcription factor MYBS3.	
Os06g0127100	AY327040	*5.15*	*4.98*	*3.09*	*4.51*	*5.03*	*1.97*		CBF-like protein.	
Os07g0545800	AY062209	*1.83*	*2.67*	*1.84*	*2.04*	*1.89*	*1.01*		Chitin-inducible gibberellin-responsive protein.	
Os07g0674800	AY297447	*3.40*	*3.82*	*1.34*	*1.39*	*2.25*	*1.85*		AP2 domain containing protein RAP2.2 (Fragment).	
Os08g0545400	CI268958	*2.14*	*4.61*	*5.24*	*5.52*	*5.21*	*5.52*		DREB1C protein	
Os09g0457900	AK067195	*4.74*	*5.59*	*2.73*	*3.14*	*3.65*	*1.70*		AP2 domain containing protein RAP2.6 (Fragment).	
Os10g0391400	AK107854	*3.69*	*4.18*	*3.81*	*3.44*	*3.83*	*1.47*		ZIM domain containing protein.	
Os10g0392400	AK061602	*2.17*	*3.24*	*2.36*	*2.66*	*2.97*	*1.28*		ZIM domain containing protein.	
Os11g0684000	AY026332	*1.02*	*2.48*	*1.73*	*1.42*	*3.04*	*1.80*		Myb, DNA-binding domain containing protein.	
**Down-regulated TFs**										
Os01g0972800	AK110625	*−2.78*	*−2.18*	*−1.86*	*−1.96*	*−2.45*		*−2.70*		WRKY transcription factor 44.
Os01g0975300	AK061823	*−1.02*	*−1.19*	*−1.73*	*−1.11*	*−1.50*		*−1.22*		Myb-related protein Atmyb5 (Myb-related protein 5)
Os03g0410000	AK110683	*−2.20*	*−1.83*	*−1.15*	*−1.37*	*−1.07*		*−1.05*		R2R3 Myb transcription factor MYB-IF25.
Os06g0112700	NA	*−1.83*	*−1.27*	*−2.47*	*−2.29*	*−1.67*		*−2.14*		Myb, DNA-binding domain containing protein.
Os06g0187000	AB037135	*−2.80*	*−3.05*	*−3.16*	*−3.24*	*−3.10*		*−3.91*		Origin recognition complex 1.
Os07g0153000	AU082861	*−2.30*	*−2.75*	*−2.28*	*−2.37*	*−1.36*		*−2.74*		ZIM domain containing protein.
Os09g0489500	AK100210	*−1.77*	*−1.10*	*−1.60*	*−1.68*	*−1.83*		*−2.34*		Ocs-element binding factor 3.2.

Log2 values are shown for different stress regimes.

### Analysis of Promoters of DEGs in CS, HS and OS

CPMs were constructed for DEGs during CS, HS and OS using Explain™ Plant 3.0 software program. This analysis aimed at analyzing the over-represented TF binding sites (F-Match) to unveil which all matrices/TF-binding sites occurs in the gene sets. CMA analysis was carried out to find the best possible pair of TFs (promoter models) to understand the underlying mechanism of coordinated regulation of functionally related or co-expressed genes. The over-represented TF binding site (F-Match) and the promoter models are presented in Data S4. Based on the constructed CPMs, it may be inferred that binding sites of specific TFs (presented in Table 3) are enriched in the promoters of DEGs. In addition, the pair combinations in the promoter models suggest a possible combinatorial effect. Among the promoters of DEGs during CS, members of AGAMOUS like family of proteins were significantly noted besides the TFs listed in Table 3 (Data S2). The promoters of the up-regulated genes during CS were enriched in ABF1. The promoters of the DEGS during HS were enriched for the HSF matrices along with ABRE-binding bZIP factor family members like ABF2, ABF3 and ABF4 and TFs like BZ8 and GBF family protein. Members of WRKY gene family of TFs were over-represented among the promoters of the DEGs during OS. The frequency of MYB family TF matrices was high among the promoters of the down-regulated genes during HS. Similar pattern was noted in case of the DEGs during OS. Overall, it was noteworthy that promoters of down-regulated genes in all the three stresses were enriched with the matrices of MYB/MYB-related gene family of TFs. High frequency of Opaque-2 (a transcriptional activator) matrices among the promoters of down-regulated genes is noteworthy. Promoter matrices noted both in CMA and F-Match analysis results for DEGs during CS, HS and OS is presented in Table 4.

**Table 3:** CPMs enriched with the highest scores in the promoters of the differentially expressed genes during CS, HS and OS.

Gene Category	Name of the Promoter Matrices	Recognized TFs
For genes down-regulated during CS	P$GAMYB_Q2/P$O2_Q2	GAMYB, MYB101, MYB33, Myb5/Opaque-2
	P$C1_Q2/P$O2_01	ATMYB5, AtMYB82, Myb3, TT2/Opaque-2
For genes up-regulated during CS	V$HSF2_01/P$ERF2_01	HSF2, HSF2A/AT2G33710, ATERF-1, ATERF13, ERF2
	P$ARF_Q2/P$P_01	ARF1, ARF2, ARF23, ARF4, ARF6, ARF8, ARF9, ETT, MP, NPH4/MYB12
For genes down-regulated during HS	P$O2_01/P$C1_Q2	Opaque-2/ATMYB5, AtMYB82, Myb3, TT2
	P$GAMYB_Q2/P$MYBAS1_01	GAMYB, MYB101, MYB33, Myb5/AtMYB81, GAMYB, MYB101, MYB33, Myb5
For genes up-regulated during HS	P$ABI4_01/V$HSF2_01	ABI4/HSF2, HSF2A
	P$C1_Q2/V$HSF2_02	ATMYB5, AtMYB82, Myb3, TT2/HSF2, HSF2A, HSF2B
For genes down-regulated during OS	P$ARR10_01/P$GAMYB_01	ARR10/GAMYB, MYB101, MYB33, Myb5
	P$O2_01/V$HSF2_01	Opaque-2/HSF2, HSF2A
For genes up-regulated during OS	P$EMBP1_Q2/V$HSF2_01	GBF1, GBF2A/HSF2, HSF2A
	V$HSF_Q6/V$HSF1_Q6	HSF, HSF1/HSF1, HSF1-L, HSF1-S, HSF1long, HSF1short

P$: Plant matrices; V$: Vertebrate matrices.

**Table 4:** Promoter matrices noted both in CMA and F-Match analysis results for the differentially expressed genes during CS, HS and OS.

Gene Category	Name of the Promoter Matrices	Recognized TFs
For genes down-regulated during CS	P$O2_01	Opaque-2
	V$HSF1_01	HSF1, HSF1-L, HSF1-S, HSF1long, HSF1short
	P$O2_Q2	Opaque-2
	P$GAMYB_Q2	GAMYB, MYB101, MYB33, Myb5
	P$ZAP1_01	WRKY1
For genes up-regulated during CS	P$ABF1_01	ABF1, ABF4
	P$CPRF2_Q2	G/HBF-1
For genes down-regulated during HS	P$O2_01	Opaque-2
	P$O2_02	Opaque-2
	P$MYBAS1_01	AtMYB81, GAMYB, MYB101, MYB33, Myb5
	P$GAMYB_Q2	GAMYB, MYB101, MYB33, Myb5
	P$GAMYB_01	GAMYB, MYB101, MYB33, Myb5
For genes up-regulated during HS	V$HSF1_Q6	HSF1, HSF1-L, HSF1-S, HSF1long, HSF1short
	V$HSF2_01	HSF2, HSF2A
	V$HSF2_02	HSF2, HSF2A, HSF2B
For genes down-regulated during OS	P$GAMYB_Q2	GAMYB, MYB101, MYB33, Myb5
	P$GAMYB_01	GAMYB, MYB101, MYB33, Myb5
For genes up-regulated during OS	V$HSF1_Q6	HSF1, HSF1-L, HSF1-S, HSF1long, HSF1short
	V$HSF_Q6	HSF, HSF1
	P$O2_02	Opaque-2
	P$OCSBF1_01	OCSBF-1
	V$HSF2_01	HSF2, HSF2A
	P$CPRF2_01	G/HBF-1
	P$EMBP1_Q2	GBF1, GBF2A
	P$WRKY_Q2	WRKY1, WRKY11, WRKY15, WRKY21, WRKY25, WRKY26, WRKY3, WRKY4, WRKY7, WRKY74

P$: Plant matrices; V$: Vertebrate matrices.

## Discussion

To understand the molecular response in rice following sub lethal stress levels that often result in adaptive response to subsequent severe stresses, transcript profilings as affected by CS, HS and OS were analyzed in rice seedlings. Our microarray based transcript profiling data showed high correlation to Q-PCR based transcript profiling data as evidenced for 23 OsHsf [31], 3 OsClpB genes [54] and genes tested in this study (Fig. S2). A large number of DEGs in response to above stresses were noted in this study. Exhaustive GO enrichment and functional classification of DEGs showed a considerable overlap for the enriched classes. DEGs were classified as specific to a given stress type or common amongst 2 or 3 stresses with respect to their expression profiles during CS, HS and OS. The specificity in the expression pattern of genes was noted also with reference to the time points of analysis. Further, genes showing induction or repression in response to all 3 stress conditions were noted. The latter class of genes may function as integrators of multiple environmental signals. Also such genes may function as co-regulators that respond to a variety of abiotic stresses and/or represent the modules (response networks) that might be involved in the cross-talk.

While transcript profilings have been analyzed to an extent in response to CS and HS in rice [33], [37], [55], [56], not much is known about how OS modulates the transcriptional dynamics in this species. This study provides a platform to compare and contrast CS, HS and OS induced transcript changes. From this study, metabolism associated with ROS appears as a central theme in the 3 stresses analyzed. This is evident from the fact that genes involved in redox homeostasis in response to OS were also differentially regulated during CS and HS. Several such genes were co-regulated during CS and OS as well as during HS and OS. HSFs and HSPs are important components of HS regulatory networks [30], [57], which were also prominently noted in DEGs affected by OS. It can thus be inferred that HSFs-HSPs regulon is a redox responsive regulatory system. Swindell *et al.* [58] noted that HSFs and HSPs represent an interaction point amongst multiple stress responsive pathways. In this study, matrices corresponding to HSF bindings sites were noted in the promoters of several DEGs in response to CS, HS and OS (Tables 3, 4) suggesting that HSFs are major players in the stress response. It has earlier been noted that HSPs are induced by a variety of stress conditions [21], [40], [59]–[63]. Wang *et al.* [64] has proposed that a cross-talk exists between HSP/chaperone and other stress responsive mechanisms in plants. Banti *et al.* [65] noted that cross-adaptation mechanisms between HS and anoxia involve HSPs. In addition, Collinet *et al.* [66] drew parallels between HS and CS responsive networks in *Drosophila*. In this report, HSFs and HSPs were found differentially regulated during CS, suggesting that both HS and CS lead to differential expression of the HS genes. Furthermore, HSPs were also shown to protect against ROS damage [67]. Importantly, absence of any gene involved in redox homeostasis in early CS (CS1 h) suggests that ROS mediated cross-talk or response occurs late in CS response. Taken together, it appears that HSF/HSP regulon may be regarded as the central regulator of plant stress responses involving ROS accumulation.

Various major transcription factor classes like DREB1/CBF, DREB2, NAC, MYB/MYC and HSFs were differentially regulated in all the 3 stress types studied. Enrichment of MADS box gene family members in HS response is a noteworthy observation. Transcripts for ZIM proteins (known to have role in plant abiotic stress response [68]) were prominently altered during stresses in this study (Table 2; Data S1). It has been observed that MAP kinases and CDPK pathways are involved in abiotic stress induced defense response in plants. However, MAP kinases are not neatly delineated into separate parallel cascades; instead they show lot of overlap and are involved in cross-talk [25], [69], [70]. As kinases were differentially expressed in all 3 stress conditions, we concur with the postulation that kinases are involved in cross-talk. We also infer that ROS and Ca^2+^ mediated gene networks may be shared to a great extent during CS, HS and OS in rice. Xuan *et al.* [71] showed that AtCaM3 is a key component of the NO pathway in HS responsive signaling cascade. According to Zhang *et al.* [72], increased cytosolic concentration of Ca^2+^ directly activates AtCaM3 and results in adaptations to HS. HS resulted in differential expression of genes involved in Ca^2+^ signaling. Overall, we show that the expression of TFs and kinases was well supported by genes for signaling components (such as proteins involved in Ca^2+^ signaling and phosphatases).

The onset of metabolic reprogramming may enable the adjustments of energy/osmotic homeostasis, as a key response to stressful regimes. Genes involved in synthesis of raffinose family oligosaccharides and others like trehalose were found enriched following stress conditions applied. Galactinol and raffinose were shown to have a role in scavenging hydroxyl radicals enabling protection of plant cells from oxidative damage by abiotic stresses [73]. Further, HS induced production of galactinol and raffinose was also shown [74]. Importantly, galactinol synthase 1 is one of the HSF target genes responsible for heat-induced synthesis of raffinose family oligosaccharides in Arabidopsis [75]. Metabolic reprogramming was apparent from several other transcript changes in this study. This observation is in concurrence to the report of Marsoni *et al.* [76]. Sakata *et al.* [77] found that endogenous auxin levels specifically decreased in developing anthers of barley and Arabidopsis under HS. HS mediated induction of auxin responsive genes (IAA29) and role of bHLH (TF PIF4) suggesting integration of auxin mediated signaling with HS response was also shown [78]. Recently, it was shown that Aux/IAA protein HaIAA27 represses transcriptional activation by HaHSFA9, which controls a genetic program involved in seed longevity and embryonic desiccation tolerance [79]. Our data indicates that auxin mediated signaling is a constituent in CS, HS and OS stress responses.

Several *ab initio* motif discovery methods have been developed and applied to gene expression data in recent years (AlignACE, [80]; REDUCE, [81], [82]). These methods strive towards the goal of finding a pattern in promoters that shows a statistically significant dependency with the observed expression levels or variables associated with these expression levels (e.g. clusters of co-expressed genes). High-throughput efforts have been placed, for instance, for the identification of transcription start sites and conserved promoter motifs in several organisms [83], [84]. Comparing the results of F-Match and CMA (Table 4), a clear pattern of over-represented HSF binding sites in the promoters of up-regulated genes during HS and OS treatment and not in CS up-regulated genes was noted. A high similarity was found between the promoter models of HS and OS responsive genes. It is evident that gene regulation is accomplished by specific combination of TFs rather than by single factor alone. For example, expression of AtHSP90-1 gene is reportedly regulated by interaction between HS and other transcription binding sites [activating protein-1 (Ap-1), CCAAT/enhancer binding protein element (C/EBP), and metal regulatory element (MRE)] [85]. It is possible that the modules noted are responsible for a function specific regulation of transcription. Overall, these TFs may form positive/negative feedback loops in the signal transduction circuits. The pair combinations in the promoter models and F-Match analysis may be explored to identify unique type of combinatorial transcriptional control.

The central role of ROS homeostasis during temperature extremes is highlighted from this study. Pretreatment of plants with stress conditions that induce ‘oxidative burst’ can trigger a protective function or immunize the plants against environmental stresses and thus could play a role in acclimatizing stress tolerance [86], [87]. In addition it was proposed that signal transduction events following various stress conditions ultimately affects common set of TFs associated with antioxidative defense enzymes [88]–[91]. Our results are largely in accordance with the above propositions. However the actual biological function(s) of large number of DEGs noted in this study needs to be validated in future.

It is likely that DEGs noted in this study are relevant for acclimation of stress tolerance. It is thus important to further unravel the relation of stress tolerance with DEGs noted in this study. We have recently noted that growth of rice seedlings following CS is different when seedlings are pre-treated or co-treated with OS along with CS and likewise, the growth of rice seedlings following HS is different when pre-treated or co-treated with OS along with HS (unpublished data). We are presently analyzing how CS and OS together as well as HS and OS together (pre-treatment as well as co-treatment) affect the gene expression in rice seedlings (manuscript in preparation). The DEGs noted in this study may have relevance in development of cross-protection to different abiotic stresses. In summary, our data highlights the global convergence and divergence of the transcriptome in response to oxidative stress and temperature extremes. Co-ordinated expression pattern and similar promoter architectures (promoter models and overrepresented transcription factors) suggest potential regulons (for instance; HSF: HSP and MYB regulon). The differentially expressed genes noted in this study might be the key players in the adaptive response of plants following sub lethal stress conditions. The data sets generated in this study may provide reference point for stress-regulated transcriptome and data mining resource for abiotic stresses.

## Supporting Information

Figure S1 **PCA plots: PCA on conditions; samples with similar scores for one or more PCA components can be considered similar in their expression profile.** Entities with high scores for a particular PCA component follow the expression pattern shown in PCA loading plots.
(TIFF)

Figure S2 **Q-PCR analysis and comparison with the microarray data for the selected TFs described in the** **Table 2** **.** The blue bars represent the Q-PCR data (relative transcript abundance).
(TIFF)

Table S1 **Details of the genes noted in network clusters.** The DEGs noted as part of the clusters are listed with BKL description. The fold changes (Log2 values) are also shown.
(DOCX)

Table S2 **Differentially expressed genes common to all the three stress conditions tested by more than 2 fold (Log2 values).**
(DOCX)

Table S3 **Specifically up/down-regulated miRNA genes.**
(DOC)

Data S1 **Detailed gene lists (described and mentioned in this study) with fold changes and hierarchical clusters (Heat maps).**
(ZIP)

Data S2 **Functional classification of DEGs.** Data S2.1–S2.6 provides the detailed functional classification of the genes for DEGs during CS, HS and OS.
(ZIP)

Data S3 **Transcript profiles of transcription factors.**
(ZIP)

Data S4 **Promoter analysis of DEGs.** Data S4.1; significantly over represented transcription factor binding sites (TFBS) in the promoters of DEGs based on F-Match analysis, Data S4.2; Match out put/TF binding sites (Rice matrices) in the promoters of DEGs based on CMA analysis. The TFs that correspond to these matrices are also listed.
(ZIP)
