# Supplementary figures and images for: Genome-Wide Transcriptional Profiles during Temperature and Oxidative Stress Reveal Coordinated Expression Patterns and Overlapping Regulons in Rice
Source: PLoS One. 2012 Jul 16;7(7):e40899. doi: 10.1371/journal.pone.0040899 (PMC3397947; doi:10.1371/journal.pone.0040899)

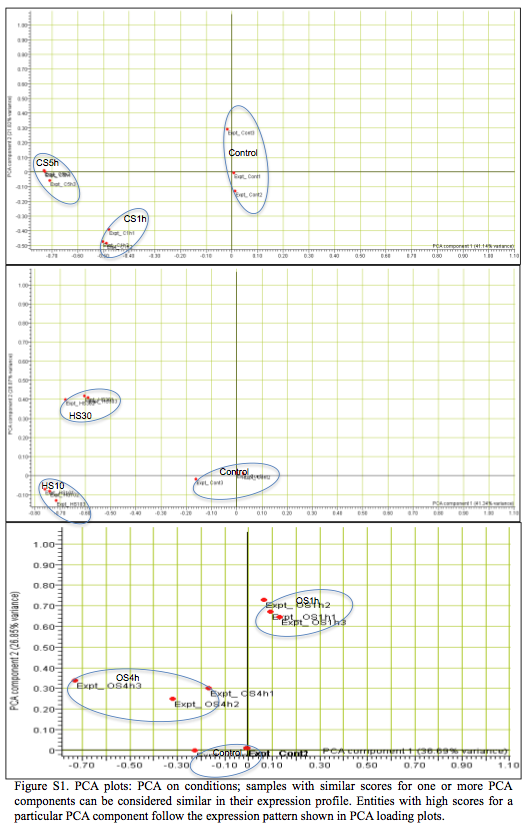

Supplement: Figure S1 — PCA plots: PCA on conditions; samples with similar scores for one or more PCA components can be considered similar in their expression profile. Entities with high scores for a particular PCA component follow the expression pattern shown in PCA loading plots. (TIFF) [file pone.0040899.s001.tiff]

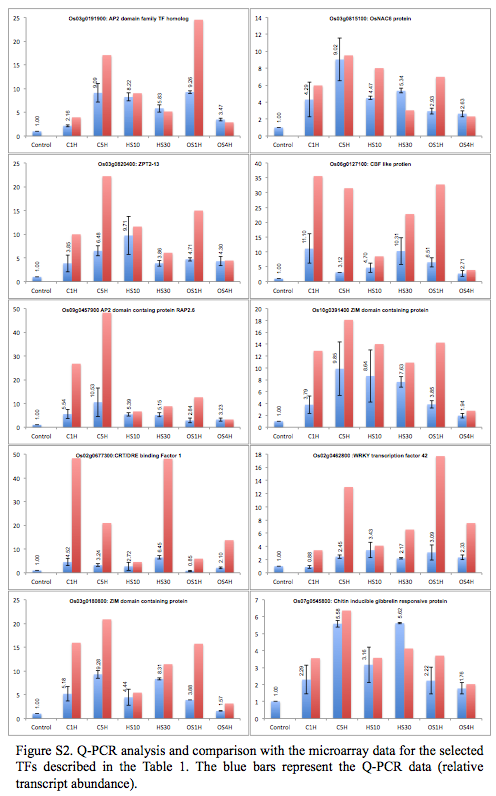

Supplement: Figure S2 — Q-PCR analysis and comparison with the microarray data for the selected TFs described in the Table 2 . The blue bars represent the Q-PCR data (relative transcript abundance). (TIFF) [file pone.0040899.s002.tiff]
