# Supplementary material for: Genome-Wide Transcriptional Profiles during Temperature and Oxidative Stress Reveal Coordinated Expression Patterns and Overlapping Regulons in Rice
Source: PLoS One. 2012 Jul 16;7(7):e40899. doi: 10.1371/journal.pone.0040899 (PMC3397947; doi:10.1371/journal.pone.0040899)
Supplement: Table S1 — Details of the genes noted in network clusters. The DEGs noted as part of the clusters are listed with BKL description. The fold changes (Log2 values) are also shown. (DOCX) [file pone.0040899.s003.docx]

Table S1. Details of the genes noted in network clusters. The DEGs noted as part of the clusters are listed with BKL description. The fold changes (Log2 values) are also shown.

| **Genes in CS Network Clusters**  **Cluster (i)** | | | | |
| --- | --- | --- | --- | --- |
| **Gene symbol** | **Gene ID/Identifier** | **BKL description** | **C1H** | **C5H** |
| CycD5-3 | Os03g0203800 | Cyclin D5-3; a D-type cyclin; binds to cyclin-dependent protein kinase (CDK) inhibitor EL2 | 1.67 | 2.43 |
| ORC1 | Os06g0187000 | Origin recognition complex 1; a putative DNA binding protein that may play a role DNA replication initiation; expressed in young leaf; highly in shoot apical meristem; and weakly in flag leaf; ear; root; and root tip | -2.8 | -3.0 |
| Os03g0302700 | Os03g0302700 | Protein with high similarity to S. pombe Apc11p; which is a ubiquitin-protein ligase; contains a C3HC4 type (RING) zinc finger | 2.16 | 1.85 |
| Os03g0645200 | Os03g0645200 | Member of the initiation factor 2 subunit family; has high similarity to a region of rat Eif2b4; which is a guanyl-nucleotide exchange factor | 1.88 | 2.6 |
| Os04g0504800 | Os04g0504800 | Protein of unknown function | 2.57 | 2.83 |
| Os09g0326900 | Os09g0326900 | Protein with high similarity to Maize eIF-5; which is a single-stranded DNA binding protein; member of the IF2B-IF5 domain family and the eIF4-gamma; eIF5 or eIF2-epsilon family | -1.7 | -2.5 |
| Os09g0452700 | Os09g0452700 | Member of the ribosomal L40e family; contains a ubiquitin family domain | 1.66 | 2.52 |
| RPA70a | Os02g0776800 | RPA70kDa subunit-a; a single stranded DNA binding protein that is highly expressed in root tips; roots; shoot apical meristem; young leaves; flag leaves; and ears; may play a role in DNA replication and repair; transcription; and cell proliferation | 1.77 | 2.87 |
| **Cluster (ii)** | | | | |
| Os03g0278300 | Os03g0278300 | Protein with high similarity to mouse Thoc4; which is a transcription coactivator that plays a role in the nuclear export of spliced and unspliced mRNAs; contains an RNA recognition motif (RRM; RBD; or RNP) | 2.38 | 2.65 |
| Os03g0646800 | Os03g0646800 | Protein containing an RNA polymerase Rpb2 domain 6 domain and an RNA polymerase Rpb2 domain 7; has high similarity to a region of S. cerevisiae RPB2; which mediates snoRNA metabolism and RNA elongation from RNA polymerase II promoter | 2.34 | 3.25 |
| Os05g0389800 | Os05g0389800 | Protein with high similarity to A. thaliana EMB2733; member of the helicase conserved C-terminal domain containing family and the DUF1605 domain of unknown function family; contains a DEAD-DEAH box helicase domain and a a HA2 domain | 1.47 | 4.47 |
| Os12g0641500 | Os12g0641500 | Member of the SMC (structural maintenance of chromosomes) C-terminal domain containing family; has low similarity to a region of structural maintenance of chromosomes 1-like 1 (human SMC1A); which is a putative ATPase required in sister chromatid cohesion | 1.65 | 2.65 |
| **Cluster (iii)** | | | | |
| MPK5/ OsMAP1 | Os03g0285800 | Mitogen-activated protein kinase 5; a multiple stress responsive MAP kinase that binds to MEK1; inhibits the expression of PR genes; mediates fungal and bacterial resistance; acts in cold; drought; and salt tolerance; and is induced by silencing of MAPK6 | 1.72 | 2.28 |
| Os01g0566100 | Os01g0566100 | Protein of unknown function; has low similarity to a region of A. thaliana ELF3; which is involved in circadian rhythm; unidimensional cell growth; and red and far-red light phototransduction | 2.01 | 2.71 |
| Os02g0787300 | Os02g0787300 | Protein with high similarity to mitogen-activated protein kinase kinase 4 (A. thaliana MKK4); contains protein kinase and protein tyrosine kinase domains | 2.4 | 3.42 |
| **Cluster iv** | | | | |
| CAM1-2 | Os07g0687200 | Protein with very strong similarity to A. thaliana CAM7; which mediates Ca^2++^-mediated signaling; contains four EF hand domains | 1.92 | 2.09 |
| CPK20 | Os07g0568600 | Protein with high similarity to A. thaliana CDPK32; which is a Ca^2++^-dependent protein kinase C that is involved in response to salt stress and abscisic acid mediated signaling; contains four EF hand domains and a protein kinase domain | 1.65 | 2.52 |
| OsCPK6 | Os02g0832000 | Protein with high similarity to A. thaliana CPK6; which mediates abscisic acid mediated signaling and regulates anion channel activity and stomatal movement; contains a protein kinase domain and four EF hand domains | 1.9 | 2.88 |
| **Genes in HS Network Clusters**  **Cluster (i)** | | | | |
| **Gene symbol** | **Gene ID/Identifier** | **BKL description** | **HS10** | **HS30** |
| ORC1 | Os06g0187000 | Origin recognition complex 1; a putative DNA binding protein that may play a role DNA replication initiation; expressed in young leaf; highly in shoot apical meristem; and weakly in flag leaf; ear; root; and root tip | -3.1 | -3.2 |
| Os02g0797400 | Os02g0797400 | Protein of unknown function | -0.4 | -3.6 |
| Os05g0111000 | Os05g0111000 | Protein containing an OB-fold nucleic acid binding and a replication factor-A protein 1 N-terminal domain and four zinc knuckle domains; has low similarity to human RPA1; which mediates protein complex assembly; recombinational repair; and DNA ligation | 2.57 | 2.08 |
| Os05g0160800 | Os05g0160800 | Member of the DNA polymerase epsilon subunit B family | -1.0 | -3.7 |
| Os05g0358200 | Os05g0358200 | Protein of unknown function | -1.0 | -3.2 |
| Os06g0506600 | Os06g0506600 | Protein with high similarity to A. thaliana UBC28; which is involved in ubiquitin-dependent protein catabolic process; contains a ubiquitin-conjugating enzyme domain | 2.35 | 2.65 |
| Os09g0483400 | Os09g0483400 | Member of the ribosomal L40e family; contains a ubiquitin family domain | 2.49 | 2.14 |
| UBC5b | Os02g0261100 | OsUBC5b; an Ubc4/5 subfamily E2-ubiquitin-conjugating enzyme that is induced in response to N-acetylchitoheptaose; interacts with EL5 and catalyzes EL5-mediated ubiquitination; may play a role in proteasomal ubiquitin-dependent protein catabolic process | 2.88 | 2.09 |
| **Cluster (ii)** | | | | |
| Os02g0757900 | Os02g0757900 | Protein with high similarity to A. thaliana AT5G10350; which interacts with polyadenylate and proteins; contains an RNA recognition motif (RRM; RBD; or RNP) | 2.58 | 2.73 |
| Os03g0646800 | Os03g0646800 | Protein containing an RNA polymerase Rpb2 domain 6 domain and an RNA polymerase Rpb2 domain 7; has high similarity to a region of S. cerevisiae RPB2; which mediates snoRNA metabolism and RNA elongation from RNA polymerase II promoter | 2.32 | 2.28 |
| Os05g0364600 | Os05g0364600 | Protein with high similarity to Arabidopsis thaliana serine-arginine protein 30.1 (A. thaliana ATSRP30.1); which is involved in mRNA splice site selection; contains two RNA recognition motifs (RRM; RBD; or RNP) | 1.82 | 2.47 |
| Os05g0367000 | Os05g0367000 | Protein with strong similarity to rat Phf5a; which is a ligand-dependent nuclear receptor transcription coactivator that acts in estrogen receptor signaling pathway and in response to hormone stimulus; member of the PHF5-like protein family | 3.84 | 3.21 |
| Os05g0389800 | Os05g0389800 | Protein with high similarity to A. thaliana EMB2733; member of the helicase conserved C-terminal domain containing family and the DUF1605 domain of unknown function family; contains a DEAD-DEAH box helicase domain and a a HA2 domain | 1.15 | 4 |
| Os07g0633200 | Os07g0633200 | Protein with high similarity to A. thaliana SCL33; which is involved in nuclear mRNA splicing; via spliceosome; contains an RNA recognition motif (RRM; RBD; or RNP) | 3.18 | 3.11 |
| **Cluster (iii)** | | | | |
| Os07g0271500 | Os07g0271500 | Protein with high similarity to A. thaliana CHS; which is a naringenin-chalcone synthase; involved in auxin polar transport; contains a chalcone and stilbene (resveratrol) synthase N-terminal domain and a chalcone and stilbene synthase C-terminal domain | -2.1 | -2.3 |
| Os07g0525500 | Os07g0525500 | Protein with high similarity to CHALCONE SYNTHASE (A. thaliana CHS); which is a naringenin-chalcone synthase that acts in anthocyanin and flavonoid biosynthesis; contains a chalcone and stilbene (resveratrol) synthase N-terminal and C-terminal domains | 2.47 | 1.86 |
| Os07g0525900 | Os07g0525900 | Member of the plant Tnp2 transposase family; contains a ubiquitin-like protein-specific protease family C-terminal catalytic domain; a chalcone and stilbene synthase C-terminal and N-terminal domains; has a region of high similarity to A. thaliana CHS | 2.62 | 2.01 |
| Os07g0526400 | Os07g0526400 | Protein with high similarity to A. thaliana CHS; which is a naringenin-chalcone synthase that acts in auxin polar transport; and chalcone biosynthetic process; contains chalcone and stilbene (resveratrol) synthase N-terminal and C-terminal domain | 2.47 | 2.48 |
| **Cluster (iv)** | | | | |
| Os04g0107900 | Os04g0107900 | Protein containing a histidine kinase-like ATPase domain; has strong similarity to a region of A. thaliana HSP81-1; which is involved in defense response to bacterium and response to heat | 9.49 | 9.56 |
| Os09g0482400 | Os09g0482400 | Protein with very strong similarity to Rice Os09g0482100; which exhibits protein kinase binding; member of the heat shock Hsp90 protein family; contains a histidine kinase-like ATPase domain | 2.38 | 1.86 |
| rHsp90 | Os06g0716700 | Heat shock protein 90 kDa; a heat shock protein that mediates salt and heat tolerance; expression is induced in root and leaf of seedlings in response to high temperature; osmotic stresses; and alkaline pH | 1.94 | 2.74 |
| **Genes in OS Network Clusters**  **Cluster (i)** | | | | |
| **Gene symbol** | **Gene ID/Identifier** | **BKL description** | **OS1H** | **OS4H** |
| Os07g0525500 | Os07g0525500 | Protein with high similarity to CHALCONE SYNTHASE (A. thaliana CHS); which is a naringenin-chalcone synthase that acts in anthocyanin and flavonoid biosynthesis; contains a chalcone and stilbene (resveratrol) synthase N-terminal and C-terminal domains | 3.84 | 0.59 |
| Os07g0525900 | Os07g0525900 | Member of the plant Tnp2 transposase family; contains a ubiquitin-like protein-specific protease family C-terminal catalytic domain; a chalcone and stilbene synthase C-terminal and N-terminal domains; has a region of high similarity to A. thaliana CHS | 4.03 | 0.65 |
| Os07g0526400 | Os07g0526400 | Protein with high similarity to A. thaliana CHS; which is a naringenin-chalcone synthase that acts in auxin polar transport; and chalcone biosynthetic process; contains chalcone and stilbene (resveratrol) synthase N-terminal and C-terminal domain | 3.65 | 1.06 |
| **Cluster (ii)** | | | | |
| ORC1 | Os06g0187000 | Origin recognition complex 1; a putative DNA binding protein that may play a role DNA replication initiation; expressed in young leaf; highly in shoot apical meristem; and weakly in flag leaf; ear; root; and root tip | -3.1 | -3.9 |
| Os04g0504800 | Os04g0504800 | Protein of unknown function | 2.6 | 1.75 |
| **Cluster (iii)** | | | | |
| Os07g0628900 | Os07g0628900 | Member of the DUF26 domain of unknown function family; has a region of moderate similarity to a region of interleukin 1 receptor associated kinase 4 (human IRAK4); which is involved in MAPKKK cascade | -3.7 | -1.6 |
| Os10g0530900 | Os10g0530900 | Protein containing a glutathione S-transferase N-terminal domain and a C-terminal domain; which may act in conjugation of reduced glutathione to various targets; has moderate similarity to A. thaliana ATGSTU8; which is involved in response to nitrate | 2.40 | 3.05 |
