# Supplementary material for: Genome-Wide Transcriptional Profiles during Temperature and Oxidative Stress Reveal Coordinated Expression Patterns and Overlapping Regulons in Rice
Source: PLoS One. 2012 Jul 16;7(7):e40899. doi: 10.1371/journal.pone.0040899 (PMC3397947; doi:10.1371/journal.pone.0040899)
Supplement: Table S2 — Differentially expressed genes common to all the three stress conditions tested by more than 2 fold (Log2 values). (DOCX) [file pone.0040899.s004.docx]

Table S2. Differentially expressed genes common to all the three stress conditions tested by more than 2 fold (Log2 values).

| **Up_All** | | | | | | | | |
| --- | --- | --- | --- | --- | --- | --- | --- | --- |
| **Gene** | **cDNA** | **CS1H** | **C5SH** | **HS10** | **HS30** | **OS1H** | **OS4H** | **Description** |
| Os03g0820200 | CI206634 | ***2.07*** | ***4.51*** | ***5.19*** | ***3.31*** | ***4.97*** | ***3.07*** | Obsolete loci |
| Os09g0341500 | AK073913 | ***3.46*** | ***5.03*** | ***2.38*** | ***3.29*** | ***3.64*** | ***2.96*** | Expressed protein |
| Os03g0820400 | AY305864 | ***3.27*** | ***4.45*** | ***3.47*** | ***2.56*** | ***3.89*** | ***2.13*** | Zinc finger DNA-binding protein, putative, expressed |
| Os08g0374600 | AK072505 | ***2.66*** | ***4.47*** | ***2.47*** | ***3.98*** | ***4.24*** | ***2.41*** | Receptor protein kinase CRINKLY4 precursor, putative, expressed |
| Os03g0325900 | Os03g0325900 | ***2.14*** | ***3.83*** | ***2.67*** | ***3.12*** | ***3.22*** | ***2.96*** | Conserved hypothetical protein |
| Os11g0668200 | CI424579 | ***2.48*** | ***3.14*** | ***4.09*** | ***3.94*** | ***3*** | ***2.3*** | Calmodulin binding protein, putative, expressed |
| Os03g0741200 | BU673046 | ***2.61*** | ***2.73*** | ***2.97*** | ***2.55*** | ***2.4*** | ***2.17*** | Obsolete Loci |
| Os04g0618400 | AK108024 | ***3.54*** | ***5.35*** | ***3.89*** | ***4.82*** | ***3.7*** | ***3.43*** | Expressed protein |
| Os01g0952900 | AK104991 | ***4.51*** | ***5.43*** | ***4.83*** | ***4.58*** | ***5.01*** | ***3.09*** | Expressed protein |
| Os07g0111100 | Os07g0111100 | ***2.43*** | ***2.16*** | ***2.05*** | ***2.03*** | ***2.4*** | ***2.22*** | Thiamine pyrophosphate enzyme, N-terminal TPP binding domain containing protein, expressed |
| Os02g0677300 | AK060550 | ***5.59*** | ***4.39*** | ***2.19*** | ***5.59*** | ***2.57*** | ***3.78*** | Dehydration-responsive element-binding protein 1A, putative, expressed |
| Os01g0678400 | AK106072 | ***2.75*** | ***4.4*** | ***2.83*** | ***2.12*** | ***2.98*** | ***2.88*** | Expressed protein |
| Os04g0563000 | CI547924 | ***5.39*** | ***6.41*** | ***3.92*** | ***2.24*** | ***2.57*** | ***2.7*** | Auxin-independent growth promoter, putative, expressed |
| Os02g0759400 | AY579411 | ***4.46*** | ***4.4*** | ***4.17*** | ***3.87*** | ***3.46*** | ***2.18*** | RING/C3HC4/PHD zinc finger-like protein, putative, expressed |
| Os01g0697700 | Os01g0697700 | ***2.9*** | ***4.61*** | ***2.73*** | ***3.48*** | ***3.36*** | ***2.14*** | Expressed protein |
| **Down_All** | | | | | | | | |
| Os10g0122500 | Os10g0122500 | ***-2.26*** | ***-2.95*** | ***-3.54*** | ***-3.99*** | ***-3.16*** | ***-5.13*** | 10-deacetylbaccatin III 10-O-acetyltransferase, putative, expressed |
