# Supplementary material for: Genome-Wide Transcriptional Profiles during Temperature and Oxidative Stress Reveal Coordinated Expression Patterns and Overlapping Regulons in Rice
Source: PLoS One. 2012 Jul 16;7(7):e40899. doi: 10.1371/journal.pone.0040899 (PMC3397947; doi:10.1371/journal.pone.0040899)
Supplement: Table S3 — Specifically up/down-regulated miRNA genes. (DOC) [file pone.0040899.s005.doc]

Table S3. Specifically Up/Downregulated miRNA genes. * Conserved genes in *indica* and *japonica* rice varieties; ** the condition in which they were differentially regulated by more than two folds (the conditions in italics refers for the downregulation); *** all predicted targets of these miRNAs in rice as per Archak and Nagaraju (2007).

| **miRNA** | **Stress Condition**** | **Targets***** |
| --- | --- | --- |
| osa-miR156e (Regulatory)*  miR156 family | Early HS | SBP-domain containing protein; |
| osa-miR159f (Regulatory)*  miR159 family | Early CS & OS | Myb like DNA binding domain protein; GAMYB; Protein Kinase APK1; Ca binding protein |
| osa-miR160e  (Regulatory & Catalytic)  miR160 family | Early HS | HSP18 Kda; Glycosyltransferase, Glutathione peroxidase, Auxin responsive factor 16; B3 DNA binding domain containing protein; Aminoacid permease family protein; Protein Kinase similar to protein kinase PVPK-1; aldo /keto reductase family protein, |
| osa-miR164c  (Catalytic)  miR164 family | Early HS | Pyruvate dehydrogenase complex dihydrrolipoamide acetyltransferase family protein, NAM protein, NB-ARC domain containing protein, NAC domain containing protein, SA induced protein 19, Protein Phosphatase 2C; Adenylate kinase; Dehydration responsive protein; Zn Finger (C3HC4-type ring finger) family protein, Metal transporter, HSP101 Kda; ubiquitin conjugating enzyme 13. |
| osa-miR166k  (Regulatory)  miR166 family | Early HS | Rolled leaf |
| osa-miR167h  (Catalytic)  miR167 family | CS | Beta-2-xylosyl transferase |
| osa-miR169a (Regulatory)*  miR169 family | Early HS; CS & OS | CCAAT-binding TF subunit B protein; Protein Kinase family Protein; Disease resistance protein |
| osa-miR169o  (Regulatory)  miR169 family | Early CS & HS | CCAAT-binding TF subunit B protein |
| osa-miR171f  miR171 family | CS; Early HS&OS | Sacre crow like TF 6 (SCL6); |
| osa-miR171h  miR171 family | CS | Sacre crow like TF 6 (SCL6); Cyclin, N terminal domain containing protein |
| osa-miR319b  (Regulatory)  miR319 family | Early HS | Myb like DNA binding domain protein, GAMYB |
| osa-miR393b  miR393 family | Early HS | LRR family protein, oxidoreductase, 20G-FE oxygenase; bHLH family protein |
| osa-miR395r  (Regulatory)  miR395 family | Early HS | NB-ARC domain containing protein |
| osa-miR398a  (Catalytic)*  miR398 family | Early OS; Early CS & OS | Superoxide Dismutase |
| osa-miR399d  (Catalytic)  miR399 family | CS; Early OS | Alcohal dehydrogenase; Ubiquitin conjugating family protein; Phosphate H^+^ symporter family protein; Endomembrane protein-70 putative (TM4 family); |
| osa-miR399h  miR399 family | Early CS | Sec23/sec24- transporter family protein; Disease resistance NBS-LRR class protein; Phosphate H^+^ symporter family protein; |
| osa-miR399i  miR399 family | Early HS | Ubiquitin conjugating family protein; Phosphate H+ symporter family protein; Got-1 like family protein |
| osa-miR399j  miR399 family | CS; Early HS | Phosphate H+ symporter family protein |
| osa-miR528  (Catalytic)  miR528 family | Early OS | Laccase; hydroxyproline rich glycoprotein family protein; F-box/LRR –MAX2 homolog protein, L-ascorbate oxidase; inositol-1,3,4, triphosphate-5/6 kinase family protein; regulator of chromatin condensation RCC1 family protein |
